# Causal beliefs about depression in different cultural groups—what do cognitive psychological theories of causal learning and reasoning predict?

**DOI:** 10.3389/fpsyg.2014.01303

**Published:** 2014-11-25

**Authors:** York Hagmayer, Neele Engelmann

**Affiliations:** Institute of Psychology, University of GoettingenGoettingen, Germany

**Keywords:** causal learning and reasoning, causal beliefs, causal model theory, lay theories of depression, cross-cultural differences

## Abstract

Cognitive psychological research focuses on causal learning and reasoning while cognitive anthropological and social science research tend to focus on systems of beliefs. Our aim was to explore how these two types of research can inform each other. Cognitive psychological theories (causal model theory and causal Bayes nets) were used to derive predictions for systems of causal beliefs. These predictions were then applied to lay theories of depression as a specific test case. A systematic literature review on causal beliefs about depression was conducted, including original, quantitative research. Thirty-six studies investigating 13 non-Western and 32 Western cultural groups were analyzed by classifying assumed causes and preferred forms of treatment into common categories. Relations between beliefs and treatment preferences were assessed. Substantial agreement between cultural groups was found with respect to the impact of observable causes. Stress was generally rated as most important. Less agreement resulted for hidden, especially supernatural causes. Causal beliefs were clearly related to treatment preferences in Western groups, while evidence was mostly lacking for non-Western groups. Overall predictions were supported, but there were considerable methodological limitations. Pointers to future research, which may combine studies on causal beliefs with experimental paradigms on causal reasoning, are given.

## Introduction

Causal learning and reasoning appears to be a universal capacity. Causal learning enables us to derive knowledge about generic causal relations from observations and actions and to test hypotheses about causal relations. Causal reasoning allows us to explain events, to diagnose causes, and to predict future events and unobserved features. For example, causal learning enables us to find out which factors cause mental distress and impairment. Causal reasoning allows us to diagnose the causes of current distress, to predict its future course, and to envision interventions which may provide relief.

Causal learning, however, requires pre-existing causal knowledge. Research in cognitive science has shown that causal learning from a limited amount of data is only feasible if there is some higher order, abstract causal knowledge that constrains the number of potential causal hypotheses (Kemp et al., 2010; Tenenbaum et al., 2011). Only if a learner has some abstract theory about which of the numerous observable variables are candidate causes and which are possible effects, a small number of observations is sufficient to derive causal knowledge, that is, generic causal beliefs that have a relevant degree of certainty. For example, to find out what factors cause digestive problems, it is important to know that (i) causes precede the symptoms and that (ii) symptoms cannot be causes even when they are observed simultaneously with or even before the condition. These abstract causal beliefs represent fundamental concepts of causality (Waldmann, 1996; White, 2006; Beller et al., 2009). In addition, higher-order, domain-specific beliefs are relevant for learning. For example, some basic medical knowledge tells us that physical injuries are not related to digestion, but nutrition and stress might be.

Like causal learning, causal reasoning is also based on causal knowledge, including higher-order theories about a domain (e.g., lay theories of illness) and specific causal beliefs about particular issues (e.g., beliefs about the causes of depression). For example, to diagnose the cause of a person's depressive symptoms, it is important to know that stress is a relevant causal factor for disease in general. When it comes to problem solving and decision making, causal knowledge may again be relevant. Sometimes purely instrumental knowledge, that is, knowledge about the consequences of actions, may be sufficient. But when no respective instrumental knowledge is available, causal knowledge may enable decision makers to choose the best course of action (Sloman and Hagmayer, 2006; Hagmayer and Meder, 2013). For example, persons with lactose intolerance usually know that taking lactase in advance prevents later digestive problems (instrumental knowledge), but only causal knowledge including at least some vague idea about the mechanism by which lactase works, allows us to infer that taking lactase after digestive problems have already occurred will give some relief.

Given that causal learning and reasoning and causal beliefs are inherently connected to each other, it may seem surprising that cognitive-psychological research often disregards people's pre-existing causal beliefs when investigating causal learning and reasoning. In order to study the underlying learning and reasoning processes, pre-existing domain-specific knowledge is usually excluded by using abstract problems or by providing participants with knowledge about new, previously unknown causal relations. These artificial scenarios ensure that participants cannot rely on pre-existing knowledge to respond to the given tasks, but have to actually engage in learning and/or reasoning based on the observed data, general notions of causality and higher-order theories. For example, in a landmark study on causal learning, Waldmann and Holyoak (1992) asked US-students to learn the relation between the disease Midosis and substances in the blood, which were either introduced as causes or as effects of the disease. Using the famous blocking paradigm, they showed participants in a first learning phase that Substance 1 was present whenever the disease was present. In a second learning phase they showed participants that Substance 2 was present whenever Substance 1 and the disease were present. In a test phase participants had to judge the likelihood of the disease given each of the substances. It turned out that participants' inferences were not only based on the observed statistical relations, but also on assumptions about the causal status of the substances. If they were assumed to be *effects* of the disease, participants considered both substances to be good predictors of the disease. By contrast, if substances were believed to be *causes* of the disease, only Substance 1 was considered to be a good predictor, while participants were unsure whether Substance 2 was a good predictor and therefore gave intermediate ratings. In other words, Substance 2 was blocked by Substance 1 only when they were assumed to be causes, but not when they were assumed to be effects.

Cognitive anthropological studies, in contrast to cognitive psychological research, often focus on studying systems of beliefs. Causal beliefs (i.e., beliefs concerning causes, consequences, interventions, and causal mechanisms) are part of the belief systems being explored. Respective research has been carried out in many cultural groups, both Western and non-Western. For example, Furnham (1988) explored people's lay theories about the causes of various medical conditions including depression, obesity, and lung cancer in the UK and elsewhere. Murdock (1980) summarized and analyzed previous anthropological work on lay medical theories in cultural groups around the globe. Despite not investigating causal reasoning *per se*, respective research showed that causal beliefs are related to other beliefs and actions. In the medical domain, for example, causal beliefs were linked to attitudes (e.g., stigma), to medical practices with respect to diagnosis and treatment, to people's expectations and predictions (e.g., prognosis of the course of an illness), and to actions (e.g., help seeking). For instance, Okello and Ekblad (2006) investigated causal beliefs about depression of the Ganda in Uganda. When witchcraft was suspected as the cause of a person's depression, the help of traditional healers was sought, while Western medicine was preferred to address somatic causes and symptoms of depression.

Thus, cognitive-psychological research on causal cognition and research on causal beliefs, which is conducted by anthropologists and other social scientists, yield important insights on causal cognition. Nevertheless, these two research traditions are still largely unconnected (cf. Beller, Bender and Waldmann's introduction to this special issue). An important and still open question is how these two types of research can best inform each other. Our aim in this paper is to provide first, tentative answers to this question. First, we will explore the predictions that can be derived from cognitive psychological theories on causal learning and reasoning for systems of causal beliefs. We will then apply these predictions to a specific test case, lay theories of depression. Expectations concerning similarities and differences between cultural groups will be laid out. In order to test these predictions, a systematic literature review of studies on causal beliefs about depression will be presented. Findings will be discussed and limitations will be pointed out. Finally, we will outline potential routes for future research, which combine studies on causal beliefs with experimental paradigms from the research on causal learning and reasoning.

### Predictions from cognitive psychological theories of causal cognition for systems of causal beliefs

Many cognitive theories have been proposed to account for causal learning and reasoning. Some of these theories have tried to reduce causal reasoning to associative learning or probabilistic reasoning, but failed to account for the abstract notions of causality that people bring to bear when reasoning causally (Waldmann and Hagmayer, 2013). Therefore, we will focus on two classes of theories, causal model theory (Waldmann, 1996; Sloman, 2005) and Causal Bayes nets (Spirtes et al., 1993; Pearl, 2000; Glymour, 2001; Griffiths and Tenenbaum, 2009), both of which assume that people base causal reasoning on abstract notions of causality and represent causal relations in their beliefs. Causal model theory is a psychological theory, which aims to describe how people actually learn and reason. Causal Bayes nets provide a rational, computational model to formally describe causal induction, knowledge, and reasoning. It models an optimal learner and perfectly rational causal thinker (Waldmann et al., 2008). Recently causal Bayes nets have been used to formally describe mental causal models and derive predictions to be tested empirically. Empirical investigations to test these predictions with Western students using artificial scenarios yielded mostly confirmatory evidence (see Rottman and Hastie, 2014, for a comprehensive overview).

Two other theories have to be mentioned first, though, as they explain when and whereof people reason causally. Norm theory (Kahneman and Miller, 1986) predicts that people will start to search for causal explanations when observing events or instances which violate norms, that is, expectations about what normally happens. Counterfactual thinking is assumed to determine the factor that caused the deviation. The abnormal conditions focus model (Hilton and Slugoski, 1986) basically makes the same prediction. Abnormal events are assumed to trigger causal analyses. Research on counterfactual thinking has provided confirmatory evidence for these predictions (cf. Roese, 1997). It also showed that counterfactual deliberations are used to establish the cause or causal contribution of a given factor to an event. Given that successful causal inquiries result in causal beliefs, these theories imply that people should hold more causal beliefs concerning abnormal than normal events. For example, people should hold more beliefs about the causes of ill-health than about the causes of good health. They also entail that people should hold similar beliefs across different cultural groups as long as the same events are considered abnormal in these groups.

**Figure 1:**
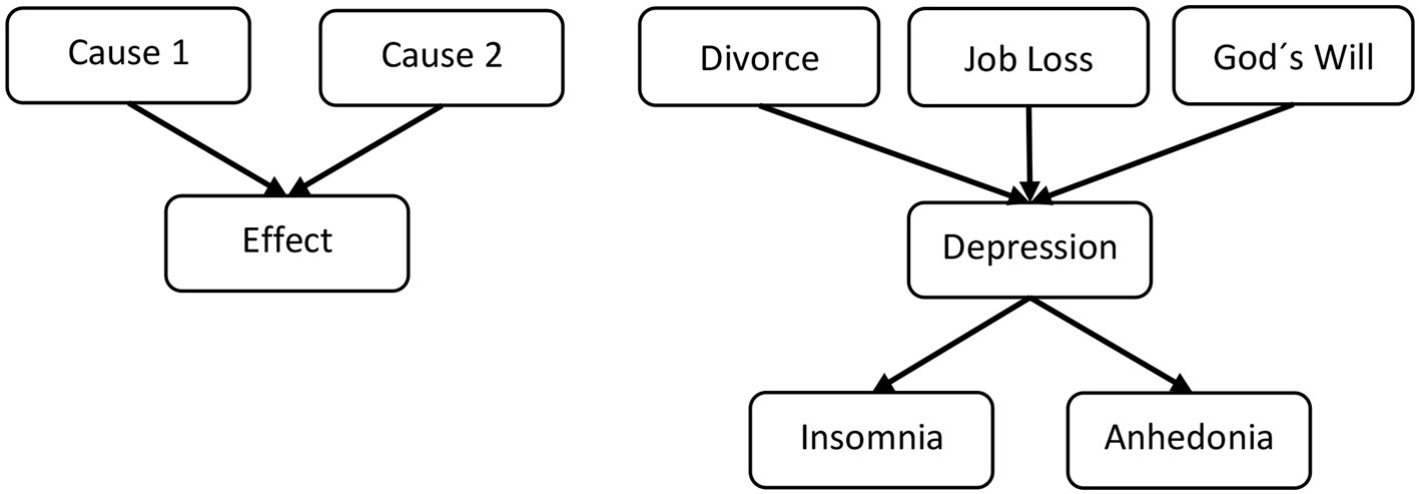
**Graphical causal models representing causal relations**. On the left hand side an abstract, generic model is depicted, on the right hand side an example for a simplified causal model of depression is presented. Nodes represent variables (events, states) and arrows represent directed causal relations.

Causal model theory (Waldmann, 1996; Sloman, 2005) and causal Bayes nets (Pearl, 2000; Glymour, 2001) assume that causal relations are represented as a set of beliefs about interconnected causal relations. In other words, causal relations in the world are represented as causal models. Causal models can be represented as graphical models, more precisely as directed acyclic graphs, which capture the asymmetry of causal relations (see Figure 1). Cause and effect variables are represented as nodes, while causal relations are represented by causal arrows capturing the assumption that there is a connecting causal mechanism by which the cause influences the effect. These theories assume that causal beliefs represent generic, directed causal relations and not merely associative relations. They also assume that causal relations are represented at the type level, that is, generic relations between types of events, rather than at the token level, that is, causal relations between individual instances. Hence, they entail that causal beliefs about an issue are complex and concern types of causes, mechanisms, and effects.

These theories, however, do not assume that people have specific knowledge about a causal mechanism, even when they assume that two variables are causally connected. Therefore, people should have more specific beliefs about the causes and consequences of a particular event or state than about the causal mechanisms by which these are related. This prediction was supported by research on explanation in Western cultural groups, which has shown that people often have only skeletal causal knowledge (i.e., knowledge about causes and effects), but do not know how the underlying causal mechanisms work (Rozenblit and Keil, 2002). Nevertheless, these theories also entail that assumptions about causal mechanisms should affect causal learning and reasoning whenever people hold such assumptions. When no connecting mechanism is known, for example, two variables should not be judged to be causally related even when they are statistically related. Respective research with Western adults (e.g., Koslowski, 1996) and children (Gopnik et al., 2004) yielded confirmatory evidence.

Hierarchical Bayes nets (Tenenbaum et al., 2011) presume that causal learning and reasoning is guided by general notions of causality and higher-order causal theories of a domain. General causal notions include the assumption that causes precede their effects and that causes can influence their effects but not vice versa. Higher-order, domain-specific causal theories are, for example, the assumption that diseases cause observable symptoms and impairments. Research on computational modeling has shown that such higher-order theories are necessary to constrain the set of causal hypotheses when learning from limited amounts of data (Tenenbaum et al., 2011). Hence this theory implies that people should have a hierarchy of causal theories. It also predicts that mental causal models for particular topics (i.e., causal beliefs with respect to a particular issue) should conform to the respective higher-order theories. For example, causal models for particular maladies (e.g., depression) should align with more general lay theories of illness held in a particular cultural group. Of course, causal learning also depends on the observed data. The induced causal model needs to explain the observations. Consequently, differences in causal beliefs about particular issues should arise either due to (i) differences in observations, or due to (ii) differences in higher-order theories. When the environment is roughly the same, then differences in causal beliefs about a particular topic should only be found when there are differences in higher-order theories.

The observability of causes and the frequency with which observations can be made should affect the beliefs being held. When causes can be directly observed, then respective beliefs can be induced rather easily from a few observations (Lagnado and Sloman, 2002; Fernbach and Sloman, 2009). Therefore, people in different cultural groups should hold similar causal beliefs about observable causes, as long as the environments in which they live, and thus the observations to be made, are the same. However, when the observed factors cannot account for the events to be explained, hidden causes have to be inferred from observable clues. As the number of observations is often rather small, there is generally a large number of hypotheses about unobserved causes that may explain the data (Steyvers et al., 2003). In this case, higher-order theories of a domain become more important, as they constrain the number of potential causal hypotheses. This entails that causal beliefs in different cultural groups should align more with respect to observable causes than with respect to unobservable causes. In addition, causal beliefs about observable causes should be less affected by higher-order theories than causal beliefs about hidden causes.

Higher-order theories, however, are not independent of the observable evidence, but they are underdetermined by the evidence (Kemp et al., 2010). Therefore, many higher order theories are compatible with a set of observations. For example, in medicine a number of highly elaborate theories of illness have been proposed (e.g., traditional Chinese medicine, Ayurvedic medicine, Western bio-medical medicine). All of these systems can account for a vast variety of observable illnesses and provide recommendations for—more or less—effective treatments. Therefore, it is not surprising that all of these systems are currently used by medical practitioners and are taught at universities. By contrast, causal models of particular issues have to directly conform to the observations made. Therefore, these models have to be revised more frequently than higher-order theories to account for new observations. This leads to the expectation that across different cultural groups, less variability should be found between causal models for particular issues than between higher-order causal theories, as long as the environment in which the groups live is roughly the same.

All theories of causal learning and reasoning assume that causal knowledge is functional. It not only allows us to explain events, but also to act and achieve goals. In other words, it not only serves epistemic, but also pragmatic goals (cf. Wellen and Danks, 2014). First, causal knowledge and reasoning can be used to make a diagnostic judgment and/or categorize a certain case. The causal model theory of categorization (Rehder and Hastie, 2001; Rehder, 2003) assumes that respective judgments are based on assumptions about the causal relations within a category. Based on the causal model of a category, the likelihood of observing a particular case can be derived. In turn, it can be inferred how likely the observed case belongs to the respective category. For example, when encountering a person showing symptoms of depression, a causal model of depression can be used to judge whether depression as an illness is present. This entails that causal assumptions and not merely observed symptoms should determine diagnostic judgments.

When it comes to decision making, people may resort to instrumental knowledge about the efficacy of certain actions, and choose the action which is most likely to yield the desired outcome. But even when people have no instrumental knowledge, causal knowledge and reasoning may prove to be very helpful as it allows us to identify the factors that are most likely to make a difference (cf. Sloman and Hagmayer, 2006). The causal model theory of choice predicts that people choose their actions based on a causal model of the given situation (Sloman and Hagmayer, 2006; Hagmayer and Sloman, 2009). To be more precise, people are assumed to first retrieve the causal model of a particular issue and instantiate it for the given case. Based on the model, simulations can be run to predict the outcomes of potential actions. By comparing the expected outcomes, the action can be selected that has the highest likelihood of achieving the most desired goal state. For example, when deciding on what to do in a case of depression, a causal model for a particular patient can be constructed based on the decision maker's causal beliefs and the information provided by the affected person. This model can then be used to predict whether social support is sufficient for the person to cope with the condition she or he is in. If the depressive symptoms are attributed to stress, social support is likely to be considered sufficient. By contrast, when the symptoms are assumed to be caused by persistent, depressogenic thinking styles, then some form of psychotherapy is likely to be judged more effective.

To sum up, theories of causal learning and reasoning, more precisely, causal model theories and causal Bayes nets, allow us to derive predictions for systems of causal beliefs and potential differences and similarities between different cultural groups. Table 1 provides an overview.

**Table 1:** **Predictions derived from causal model theories and respective research for systems of causal beliefs in general and beliefs about depression in particular**.

**Assumptions of causal model theories**	**Predictions for systems of causal beliefs**	**Predictions with respect to lay theories of depression**
Causal reasoning is triggered by unexpected, abnormal events (Hilton and Slugoski, 1986; Kahneman and Miller, 1986).	Causal beliefs concern abnormal conditions more often than normal events or conditions.	Depression is a frequent, but abnormal condition. Therefore, people across different cultural groups should hold causal beliefs about depression.
Causal beliefs represent directed, generic causal relations among cause and effect variables (Waldmann, 1996).	Causal relations are not only represented on the token level as relations among particular instances, but also as causal laws, i.e., generic causal relations, on a type level.	People across different cultural groups should have assumptions about causal factors that generally lead to depression.
Beliefs about individual causal relations are integrated into more complex causal models (Waldmann, 1996; Sloman, 2005).	Causal beliefs about a particular issue should form complex causal models.	People across different cultural groups should have interrelated beliefs about the causes, symptoms and consequences of depression.
Mechanisms are represented by mechanism placeholders, which represent merely the presence of an interconnecting mechanism (Pearl, 2000; Glymour, 2001).	Causal mechanisms are assumed to be present or absent. Knowledge about causal mechanisms is vague, often no details are known.	People across different cultural groups should have better knowledge about causal factors relevant for depression than knowledge about the underlying causal mechanisms.
Higher-order theories are necessary to induce causal models for a particular issue (Tenenbaum et al., 2011).	Causal models for specific issues conform to higher-order theories.	People across different cultural groups should possess higher-order theories, which inform models of depression. Causal beliefs about depression should align with these higher-order theories.
Higher-order theories are underdetermined by observable evidence (Kemp et al., 2010). Causal models of a particular issue have to directly conform to observations.	Many different higher order theories might be held and applied to a particular issue.	Higher order theories may deviate between different cultural groups. Higher order theories informing causal models should deviate more strongly than causal models of depression.
	Causal models should align whenever observations are similar.	
Observed causal relations in the world are the basis for the induction of causal beliefs. Inferred causal relations are as simple as possible to account for the observations made (Lagnado and Sloman, 2002; Fernbach and Sloman, 2009). Hypotheses involving hidden causes are generally underdetermined by the observed data (Kemp et al., 2010).	Causal relations involving directly observable variables are easier to learn than causal models involving hidden variables that need to be inferred.	Causal models with respect to directly observable causes and effects should be similar in different cultural groups given that the environments in which they live are similar.
	Hidden causes are only inferred when observations require to do so.	As observable causes do not fully account for depression, people across different cultural groups should have assumptions about hidden factors that contribute to depression.
	There is less agreement on hidden than observable causes.	People from different cultural groups should agree more on observable causes than hidden causes of depression.
**Assumptions concerning the usage of causal beliefs**	**Predictions**	**Predictions with respect to depression**
Categorization is based on beliefs about the causal structure underlying a category (Rehder and Hastie, 2001).	Depending on assumptions about the underlying causal structure, the same instances may be categorized differently.	Depending on assumptions about the causes of depressive symptoms and depression as an illness, the same patient may be diagnosed as medically ill or not. Patients should be more likely to be diagnosed as ill when they present with symptoms that are causes of other symptoms (e.g., depressive thinking style) or symptoms that are caused by many other symptoms (e.g., high level of distress).
Diagnosis is based on assumptions about causal structure underlying an illness (Kim and Ahn, 2002).		
Judgments are based on causal knowledge when respective knowledge is available (Garcia-Retamero and Hoffrage, 2006; Krynski and Tenenbaum, 2007; Kahneman, 2011).	Causal beliefs may bias judgments when probabilistic instead of causal judgments are requested; causal knowledge may support probabilistic judgments by giving meaning to probabilistic information and allowing decision makers to integrate the information into a causal model representation.	Causal beliefs may contribute to the over-diagnosis of depression, when the typical symptoms and causal factors are present, despite a low base rate in the respective groups of patients.
		Causal beliefs may also lead to an under-diagnosis of depression, when depressive symptoms are explained away as normal reactions to transient conditions or specific events.
Decisions on actions are based on causal or instrumental knowledge (Hagmayer and Sloman, 2009; Hagmayer and Meder, 2013).	Decision makers use causal knowledge to infer the consequences of novel options. Choices are based on the predicted causal consequences.	Persons across different cultural groups should take their beliefs about the causes of depression into account, when rating and/or choosing a treatment for depression. Therefore, preferences should agree with causal beliefs.

### Systems of causal beliefs—depression as a case study

Lay theories of depression are an interesting case to test the predictions derived in the previous section. Depression is a mental disorder that has a substantial prevalence in every country around the globe investigated so far, with rates ranging between 7% in Japan and round about 20% in the US and Western Europe (WHO, 2013). It also creates a significant burden to patients and their relatives (WHO, 2013). Second, there are higher-order theories that may inform models of depression. These higher order theories encompass theories of illness, which have been found in all investigated cultural groups (Murdock, 1980), theories of the mind (i.e., lay theories of psychology), and/or theories of mental distress (Sheik and Furnham, 2000). Third, depression is characterized by a set of directly observable symptoms, which allow lay people to identify the illness. It presents with psychological symptoms (depressed mood, anhedonia, and reduced energy) and somatic symptoms (loss of appetite and weight, sleep problems, digestive problems). Although there are differences in how patients from different cultural groups first present themselves, the same symptoms are usually described when inquired about (Kirmayer, 2001; Bhugra and Mastrogianni, 2004). Forth, many different causes and risk factors have been established for depression, including biological, psychological, social, and economic factors (NICE, 2009). Some of these factors are directly observable (e.g., poverty, marital problems), while others are not (e.g., physiological parameters, genetic predisposition). In addition, depression sometimes seems to result from particular events (e.g., post-partum depression, depression after stroke), while in other cases there seems to be no specific causal trigger. Thus, lay people should be able to learn about the observable causes and they should infer hidden causes to account for the cases in which there is no observable cause. Fifth, different types of interventions have proved to be effective for depression, including pharmacological treatments, psychotherapy and—in cases of mild to moderate forms of depression—many types of psycho-social interventions and activities on behalf of the patient (cf. NICE, 2009). These findings entail that at least mild to moderate cases could be successfully addressed by non-Western, non-bio-medical treatments. Hence even lay people in non-Western cultural groups having difficulties to access Western forms of treatment should be able to learn something about effective treatments for depression. Because of these reasons, depression seems to be an appropriate test case to investigate the predictions of theories of causal learning and reasoning for systems of causal beliefs across cultures.

Table 1 (right hand column) summarizes the specific predictions for the case of depression. Across different cultural groups people should hold generic beliefs about the causes of depression, and beliefs about observable causes should be similar as long as the environment in which people live is roughly the same. In consequence, treatment preferences should tend to align across different groups when depression is attributed to these observable causes. Differences between cultural groups are expected when environments differ. Differences despite similar environments are expected for: (i) Higher-order theories, which inform causal models of depression, (ii) assumptions about hidden causes of depression (which are informed by higher-order theories), and (iii) treatment preferences when hidden causes are assumed to be responsible for the observable symptoms. The literature review presented in the next section will show whether these predictions are supported empirically.

## Methods

We conducted a systematic review of the literature on lay theories of depression following the methodology used in the medical sciences (cf. Glasziou et al., 2001). It is important to note that none of the studies reviewed here were conducted to test the predictions derived in the previous sections (cf. Table 1). Studies in general aimed to investigate causal and non-causal beliefs about depression and to relate these beliefs to treatment preferences.

### Search strategy

Three databases (Embase, Medline, and Psychinfo) were searched using the following search terms: Depress^*^ AND (explanatory model OR illness perception OR caus^*^ model OR caus^*^ belief^*^ OR lay theory). All publications up to September 2012 were considered. Five-hundred-eighty-six papers were found after removing duplicates. By screening titles and abstracts, papers obviously not meeting the previously specified inclusion criteria (see Table 2) were excluded, which reduced the number of publications to 55. These papers were read and reference lists were screened for further potentially relevant publications. Seven further publications were identified this way. Of these 62 studies, 36 met the inclusion criteria, while 26 were excluded; three papers (Patel, 1995; Lobban et al., 2003; Angermeyer and Dietrich, 2006) for being reviews, the other papers for presenting no or only incomplete statistical analyses of collected data.

**Table 2:** **Inclusion and exclusion criteria**.

**Inclusion criteria**	**Exclusion criteria**
Design: Empirical study investigating causal beliefs with respect to depression (original research)	Case studies concerning a single or very few individuals, reviews, narrative accounts
	Publications not presenting original research
Participants: Lay-people including patients and their relatives, single or multiple cultural groups	Studies on causal beliefs with respect to mental distress or mental disorders in general
Method: Systematic assessment of causal beliefs through interviews following a protocol or standardized questionnaires	Studies with mental health professionals: e.g., physicians, psychiatrists, nurses, healers
Results: Presentation of quantitative results on causal beliefs: rating or ranking of importance of causes or percentage of persons endorsing each causal factor	Studies presenting qualitative results only, i.e., lists of potential causes without further quantitative information
	Studies presenting incomplete quantitative results

We decided not to include publications presenting only qualitative results concerning causal beliefs, because participants from all investigated cultural groups tended to assume a large variety of potential causes. Hence, differences between groups could hardly be judged. Only quantitative data allowed us to rank order categories of causes and thereby assess similarities and differences between groups systematically. However, this decision favored non-anthropological over anthropological studies, which tended to be more qualitative in nature (e.g., Kleinman, 1977). This issue is further discussed in section Limitations.

### Analysis of selected papers

All papers were analyzed using a pre-defined scheme (see Table 3). First the samples were classified into Western (W) and Non-Western (NW). Some of the non-Western groups were investigated in Western countries (e.g., Chinese Americans or Yoruba people from Nigeria living in the UK). Participants were classified as general population (G), patients (P), relations of patients (RP), students (S) and others (O) (e.g., members of self-help organizations and other specific groups of people). Sample sizes of respective groups are given in Table 3. Methods used to diagnose patients were classified into questionnaires (Q), and/or interviews (I). Some studies presented participants with case vignettes describing persons with depression. In most studies, a single vignette was used describing typical psychological and somatic symptoms of depression according to the International Classification of Disease (WHO, 2010) or the Diagnostic and Statistical Manual (DSM IV, APA, 2000). Methods used to investigate the conceptualization of depression as an illness vs. no illness or as a biomedical vs. mental illness were also classified into interviews (I) or questionnaires (Q). Frequently explanatory model interviews based on Kleinman (1977, 1980) were conducted. Popular questionnaires were *Reasons for Depression* (Addis et al., 1995), and the *Illness Perception Questionnaire* (IPQ), which was adapted to depression (Brown et al., 2001). Results with respect to four variables were assessed: (i) the conceptualization of depression, (ii) beliefs about the causes of depression, (iii) preferences with respect to treatment, and (iv) relations between conceptualization and assumed causes on one hand and treatment preferences on the other hand. Not all of these variables were measured in all studies. Beliefs about causes were rank-ordered based on either the frequency with which a particular cause was mentioned by interviewees or the ratings given in questionnaires. The same procedure was used for treatment preferences.

**Table 3:**
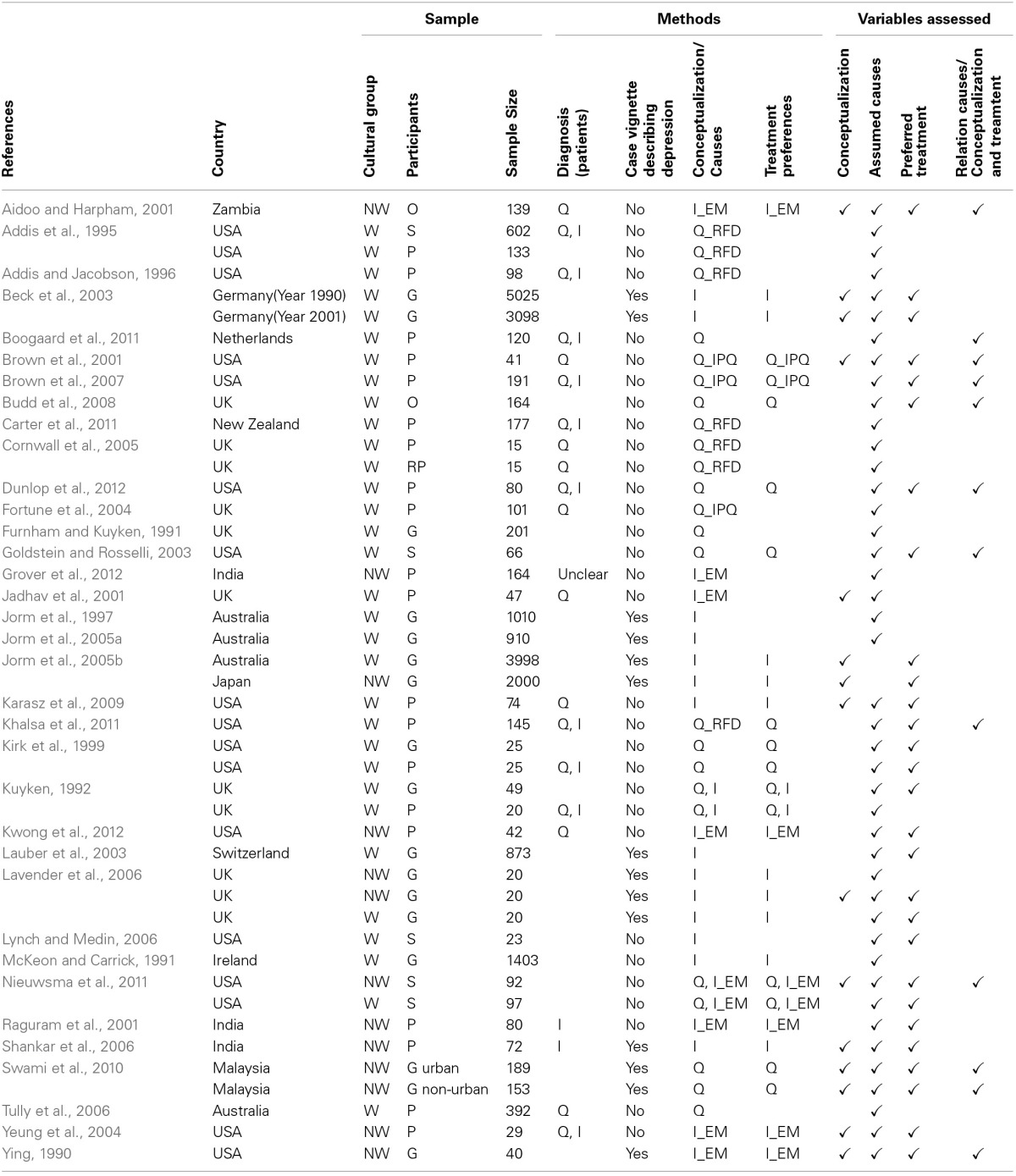
**Overview of publications meeting inclusion criteria and description of methodological details of studies**.

*Note: Participants were classified as general population (G), students (S), patients (P), relatives/spouses of patients (RP) or other (O). Cultural groups were classified as Western (W) and non-Western (NW). Methods used to diagnose patients were classified into questionnaire (Q), clinical interview (CI) or other (O). Methods used to assess conceptualizations, assumed causes and preferred treatments were classified into interviews (I) or questionnaires (Q), Explanatory Model interviews are marked as I_EM, Reasons for Depression questionnaire studies by Q_RFD and Illness perception Questionnaire studies by Q_IPQ. Check marks indicate that the respective variable was investigated*.

To enable comparisons between cultural groups, assumed causes were then classified into five categories: (i) stress (i.e., environmental factors stressing the person); (ii) personality and psychological causes; (iii) biological, (iv) supernatural, and (v) traditional causes. The classification of causes into natural vs. supernatural causes was adapted from Murdock (1980), who used the same differentiation for causes of medical illnesses. Typical examples for stress were economic hardship, marriage problems, work overload and career failure; examples for personality and psychological causes were thinking too much, lack of willpower and low resilience, examples for biological causes were chemical imbalances in the brain, genetic factors and “nerves,” and examples for supernatural causes were witchcraft, spirits and god's will. Traditional causes were causes according to non-Western medical theories. Such theories are found in India (Umma, Siddha, and Ayurvedic medicine) and in China (traditional Chinese medicine). Treatments were classified into five categories: (i) psychological treatment (e.g., psychotherapy, counseling); (ii) social support (i.e., non-professional support by family and friends); (iii) bio-medical treatment (e.g., antidepressant medication); (iv) religion or supernatural practices (e.g., praying, rituals against witchcraft), and (v) non-Western medicine or alternative treatment (e.g., Ayurvedic treatments, yoga).

## Results

### Descriptive results

It is not possible—and not necessary for the aim of this paper—to present all data with respect to conceptualizations, assumed causes and preferred treatments here. Table 4 exemplifies the causes and treatments ranked highest in the studies on non-Western cultural groups. Note that categories of causes and treatments presented in Table 4 were adopted from the respective authors. Hence they do not necessarily align with each other. A wide variety of causal factors were mentioned within and between groups. Some of these factors were culture-specific (e.g., karma in India, or ancestral spirits in sub-Saharan Africa) while others were found in virtually all groups (e.g., stress due to family issues and economic hardship).

**Table 4:** **Overview of beliefs with respect to depression in non-Western cultural groups**.

**References**	**Sample**			**Variables assessed**			
	**Cultural groups**	**Participants**	**Sample Size**	**Conceptualization**	**Assumed causes**	**Preferred treatment**	**Relation causes/Conceptualization and treatment**
Aidoo and Harpham, 2001	African: Zambian	O	139	Problem of the mind, no health-related problem	(1) Problems of the mind (unhappiness, sleep disturbance, headache)	No treatment	Reported relation (no statistical analysis): Conceptualization as no medical problem coincides with high preference for no treatment
					(2) Poverty and resulting worries		
					(3) Mood swings		
					(4) Satan, witchcraft, God		
Lavender et al., 2006	African: Yoruba	G	20	No agreement on whether person described in vignette was ill or not	(1) Magic, evil spirits, devil	(1) Religion	Reported relations (no statistical analysis): Belief in Magic/ Witchcraft as cause was associated with religious activities or witchcraft as treatment
					(2) Family problems	(2) Doctors or nurses	
					(3) Problems with partner/breakup	(3) Friends and neighbors	
					(4) Financial problems	(4) Herbalist	
							No belief in medical cause was associated with preference for no medication
Lavender et al., 2006	Asian: Bangladeshi	G	20		(1) Family problems	(1) Doctors or nurses	
					(2) Financial problems	(2) Family support	
					(3) Problems with spouse	(3) Friend support	
					(4) Worries about responsibilities	(4) Addressing the cause	
Grover et al., 2012	Asian: North Indian	P	164		Reported on spontaneously:		
					(1) Psychological causes		
					(2) Social causes		
					(3) Karma, deed, heredity		
					Reported on probing:		
					(1) Karma, deed, heredity		
					(2) Psychological causes		
					(3) Social causes		
					(4) Will of God		
Nieuwsma et al., 2011	Asian: North Indian	S	92		(1) Failure	(1) Social support	
					(2) Unfulfilled expectations	(2) Problem-focused coping	
					(3) Family issues	(3) Meditation	
					(4) Stress/Anxiety	(4) Professional treatment	
Shankar et al., 2006	Asian: Indian	P	72	Worries about life's problems, thinking too much and worries about physical health	(1) Physical disease	(1) Medication	Reported relation (no statistical analysis): Assumption of physical disease was associated with preference for medical treatment
					(2) No physical disease	(2) No treatment	
						(3) Native healing	
Raguram et al., 2001	Asian: South Indian	P	80		Reported spontaneously:	(1) Private allopath	
					(1) Social Causes	(2) Government allopath	
					(2) Medical Causes	(3) Pharmacy	
					(3) Weakness of Nerves	(4) Vow, fast, prayer, sacrifice	
					(4) Psychological Causes		
					Rated as most important:		
					(1) Weakness of Nerves		
					(2) Stress, loss, shock		
					(3) Mind, thoughts, worries		
					(4) Marital problems		
Swami et al., 2010	Asian: Malayan, rural	G	189	(1) Emotional stress	(1) Stress, pressure	(1) Counseling	Correlation between assumption of stress as a cause of depression and preference for treatment
				(2) Depression	(2) Destiny, God	(2) Psychiatrist, psychologist	
					(3) Biological causes	(3) Holiday	
					(4) Environmental causes	(4) Social support	
Swami et al., 2010	Asian: Malayan, urban	G	153	(1) Depression	(1) Stress, pressure	(1) Psychiatrist, Psychologist	
				(2) Emotional Stress	(2) Biological causes	(2) Counseling	
					(3) Environmental causes	(3) Religion, prayer	
					(4) Destiny, God	(4) Social Support	
Jorm et al., 2005b	Asian: Japanese	G	2000	(1) Psychological/ Mental/Emotional problems		(1) Talking with friends and family	
				(2) Stress		(2) Counselor	
				(3) Depression		(3) Psychiatrist	
				(4) Mental Illness		(4) Doctor	
Kwong et al., 2012	Asian: Chinese	P	42		(1) Life stress	(1) Lay help (self, friends, relatives)	
					(2) Psychological causes	(2) General health services (pharmacy, doctor, hospital)	
					(3) Medicinal causes	(3) Alternative treatment by provider (acupuncture, herbal/traditional healers)	
					(4) Traditional causes	(4) Alternative self-treatment	
Yeung et al., 2004	Asian: Chinese	P	29	(1) No psychiatric disorder	(1) Stress or psychological factors	(1) General hospital services	
				(2) Psychiatric condition	(2) Magical, religious, supernatural factors	(2) Lay help	
					(3) Medical problems	(3) Alternative treatment	
					(4) Traditional beliefs	(4) Spiritual treatment	
Ying, 1990	Asian: Chinese	G	40	(1) Psychological Problem	(1) External stress	(1) Help by psychologist or general practitioner	Assumed cause psychological: 30% seek professional help (almost all by psychologist), 30% seek non-professional help, 39% seek self-help.
				(2) Physical problem	(2) Interpersonal factors	(2) Help by family and friends	
					(3) Immigration	(3) Self-help	
					(4) Physical factors		
							Assumed cause physical: 75% seek professional help (mostly by GP), 17% seek non-professional help, 8% seek self-help

*Note: Assumed causes and preferred treatments were included when a majority of participants endorsed them or rated them above the midpoint of the respective scale. Presented categories of causes and treatments were developed by the respective authors*.

Similarities and differences within and between cultural groups became more apparent when causes and treatments were re-classified using the same category scheme and categories were rank-ordered. Table 5 shows the respective results for both Western and non-Western cultural groups. The overall rank order of cause categories was very similar across cultural groups. On average, stress due to environmental factors (e.g., family or job-related issues) was considered to be the most important cause, followed by psychological causes, biological causes and supernatural causes, although only one study on Western groups investigated the last category. But there was substantial variation between cultural groups, especially with respect to psychological, biological and—for non-Western cultural groups—supernatural causes. Some of these variations may be due to methodological differences (e.g., the specific causes inquired about or the setting in which the study was conducted); others probably reflect actual differences in beliefs.

**Table 5:**
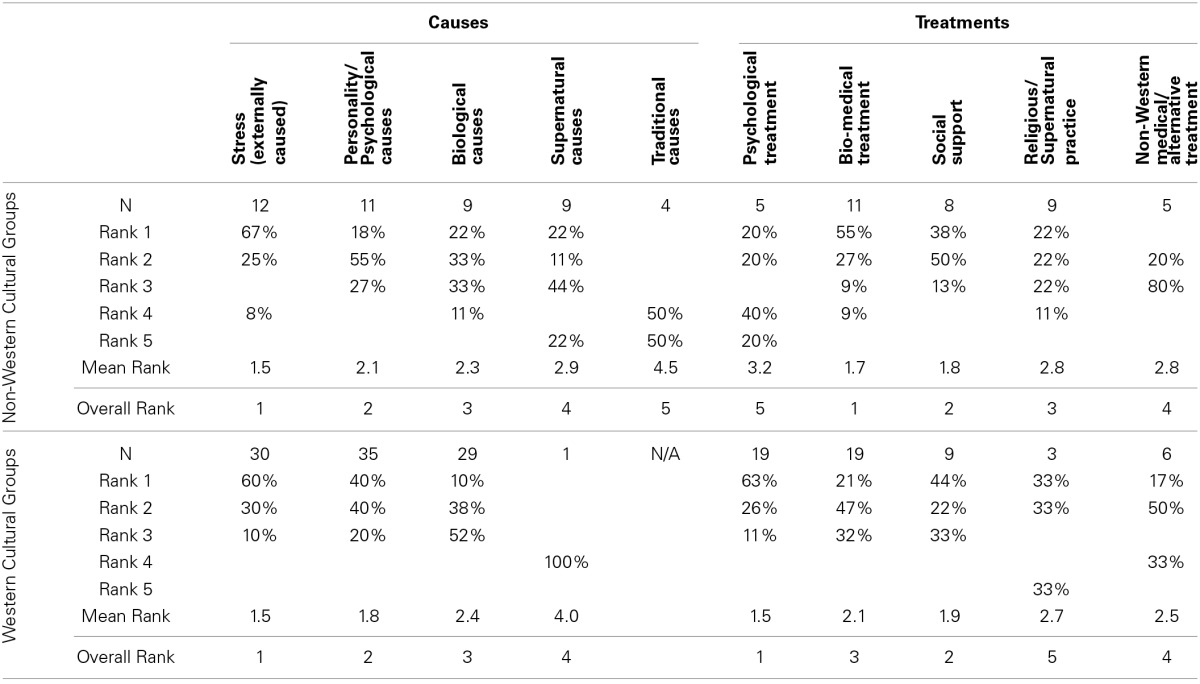
**Analysis of rank orders of assumed causes and preferred treatments in non-Western and Western cultural groups**.

*Note: Overall ranks were based on mean ranks*.

More disagreement was found with respect to treatment preferences (see Table 5 right hand side). Western cultural groups preferred psychological treatments (mostly psychotherapy) and social support over bio-medical treatments, which is in line with their causal beliefs that stress and psychological causes contribute the most to depression. A majority of non-Western groups preferred bio-medical treatments followed by social support. Religious and supernatural practices came in third ahead of traditional treatments and psychotherapy. These results, however, have to be put in context. Psychological treatments are often unavailable and therefore little known to people in non-Western countries. In addition, a number of studies—especially studies on patients—were conducted in clinics offering bio-medical treatments, which may bias participants' evaluations.

### Results concerning specific predictions

The first prediction was that people across different cultural groups should have causal beliefs about depression, because it is a frequent, but abnormal condition that affords an explanation. A vast majority of people had assumptions about the causal factors that lead to or contribute to depression. Only 0–7% claimed that they did not know about the causes. Participants tended to have more difficulties to provide a cause when depression co-occurred with psychotic symptoms (Swami et al., 2010). This is in line with other studies showing that people know less about more severe forms of mental illness. For example, a review on lay theories of schizophrenia (Angermeyer and Dietrich, 2006), found that between 5 and 15% of respondents had no respective knowledge.

The second prediction was that people across different cultural groups should have assumptions about generic causal factors for depression and that these beliefs form complex causal models. More than 90% of participants in interview studies named more than one cause of depression. The same is true for participants responding to questionnaires which endorsed more than one causal factor as relevant for depression in general or for a specific case. Hence, people seem to have complex causal beliefs regardless of cultural background.

The third prediction was that people should have more and more specific beliefs concerning causal factors than causal mechanisms. The results of the reviewed studies hardly allow us to make an informed judgment at this point. This is mostly due to methodological limitations. Both questionnaires and interviews inquired about causes of depression. Therefore, it is not surprising that respondents hardly mentioned specific causal mechanisms and gave no details about these mechanisms. One exception was cognitive mechanisms referring to rumination or thinking too much, which were reported by studies on Western and non-Western groups. Another exception was vague descriptions of Western and non-Western physiological processes in some of the questionnaire studies (e.g., chemical imbalances in the brain or humoral imbalances), which tended to be endorsed by some responders.

The fourth set of predictions concerned higher-order theories, which should inform causal models of depression. None of the studies reviewed here directly investigated higher-order theories and their implications for causal models of depression in different cultural groups. Some indirect evidence, however, comes from Indian studies, which often identified karma as a relevant cause of depression (see Table 4). Karma has to be considered an abstract theory (karma-deed-heredity), which accounts for many events and conditions including mental and other illnesses. Thus, Indian participants seem to have used this higher-order theory to causally explain cases of depression. There is at least one study outside this review, which directly addressed the question of higher-order theories. Patel (1995) investigated how lay theories of mental illness are shaped by abstract causal beliefs in sub-Saharan Africa. General assumptions held by people in this area seem to be that (i) all things and events have a cause with a greater power than the event/thing itself and (ii) that all events with high importance or impact are also caused by an intentional agent. It is believed that spirits (ancestral and others) and witchcraft can cause or at least influence events. While proximate causes are believed to explain how an event was generated, only ultimate causes are assumed to explain why a certain event happened. These general notions about causation and causal explanation explain why people in sub-Saharan Africa assume mental illnesses to be caused by social, economic, and/or biological factors (proximate causes) and supernatural causes like spirits and witchcraft (ultimate causes) at the same time.

The fifth prediction was that higher-order theories of different cultural groups should deviate more from each other than causal models of depression. Again no direct evidence is available at this moment. There are some clues, however, that may support this prediction. Academic medical theories (e.g., Western bio-medical vs. Ayurvedic vs. traditional Chinese medicine) and lay theories of illness deviate very profoundly from each other (Murdock, 1980), while the results presented here point toward a considerable amount of agreement about the causes of depression.

The sixth prediction was that people across different cultural groups would have assumptions about hidden, not directly observable causes. In virtually all studies participants assumed biological and/or supernatural causes of depression. Biological causes include physiological, nervous, and/or genetic factors or processes. These causes are not directly observable by lay people. Supernatural causes are generally assumed to be not directly observable, although they might be considered directly perceivable in some cultural groups. There seems to be some agreement, however, that the presence of a supernatural cause in a particular case has to be inferred from observable clues.

The seventh prediction was that participants from different cultural groups should agree more on observable causes than hidden causes. This prediction seems to be supported by the results shown in Table 5. Stress due to environmental factors was endorsed by all investigated cultural groups as the most important type of factor. These environmental factors are easily observable. The second most important factors were personality and psychological causes, which can be assessed through communication, followed by biological and supernatural causes. The order was the same for Western and Non-Western groups although there were differences within groups.

A number of predictions were derived concerning the usage of causal beliefs for categorization, diagnostic reasoning, judgment and decision making. Unfortunately none of the studies reviewed here directly investigated the impact of causal beliefs on categorization and diagnostic reasoning. In order to do so, participants would have to be presented with several, especially constructed cases and several judgments would have to be collected. Respective research methods exist and have been successfully used to investigate how causal assumptions of Western mental health professionals and students affect diagnostic judgments and decision making (e.g., Kim and Ahn, 2002; DeKwaadsteniet et al., 2010). For example, Kim and Ahn (2002) asked their participants (students and psychologists) to describe how the diagnostic indicators of various mental disorders are causally related to each other. Based on the individual causal models, they constructed case vignettes of patients which had symptoms that were either causes of other symptoms, effects of other symptoms, or were causally not related to other symptoms. It turned out that patients with symptoms being causes of other symptoms were judged as more likely to have the disorder than patients showing symptoms being effects or symptoms being causally unrelated. This finding is surprising, because Western mental health professionals are trained to consider all diagnostic indicators as equally important (cf. DSM IV, APA, 2000).

One important prediction of causal model theories of decision making is that people should take causal beliefs into account when deciding on actions (e.g., Sloman and Hagmayer, 2006). The relation between causal beliefs and ratings of treatments were investigated by statistical methods in 11 of the studies reviewed here. For the Western cultural groups, several studies found statistically significant relations. Dunlop et al. (2012) showed that people who attributed depression to chance or fate prefered to refrain from treatment. People who conceptualized depression as an emotional illness preferred cognitive behavioral therapy (CBT) over medication, while those who considered it a physical illness preferred medication. Khalsa et al. (2011) reported a relation between beliefs in biological causes and a preference for medication, and a relation between beliefs in childhood causes and a preference for psychotherapy. McKeon and Carrick (1991) found a positive correlation of beliefs in biological causation and perceived helpfulness of medication. Budd et al. (2008) reported the same finding. By contrast, Goldstein and Rosselli (2003) found that a belief in biological causes was related to a preference for CBT. Brown et al. (2007) showed that a belief in bio-medical and environmental causes was related to perceiving less control over the condition. Brown et al. (2001) found that people who assumed interpersonal difficulties to be an important cause adhered less to medical treatments. Boogaard et al. (2011) reported that people, who believed in childhood issues and intra-psychic fears as causes, tended to be in treatment for a longer period of time. Two studies even found a relation between causal beliefs and treatment outcomes (Addis and Jacobson, 1996; Carter et al., 2011). For example, Carter et al. (2011) showed that patients who believed that interpersonal conflicts are the cause of their depression profited more from interpersonal therapy than from cognitive behavioral therapy.

Only two studies on non-Western cultural groups directly investigated the relation, while others merely claimed their presence (see Table 4). Swami et al. (2010) found small but significant correlations among beliefs and ratings of treatments, but they tended to vary considerably between urban and rural Malay people. Those who believed more in external causes tended to rate rest and change of diet as more effective, while those who believed more strongly in supernatural causes endorsed religion as a treatment more than others. Ying (1990) found for a Chinese American sample that 30% of those who assumed psychological causes sought professional help from psychologists, while 75% of those who believed in physical causes looked for help from a physician. Other studies pointed out that (i) a belief in supernatural causes was related to respective activities and treatments (Lavender et al., 2006) and that (ii) a belief in a medical illness was related to a preference for bio-medical treatments (Aidoo and Harpham, 2001; Shankar et al., 2006). The summary presented in Table 5, however, indicates a discrepancy between the moderate belief in biological causation and the strong preference for bio-medical treatment. As pointed out above, there are several possible explanations for this finding. Patients may have tried other forms of treatment, being more consistent with their beliefs, before resorting to bio-medical treatment. Another might be that other services, especially psychotherapy or counseling are not available. In addition, the costs of different forms of treatment may have affected preferences beyond causal considerations.

In sum, many of the predictions derived from causal model theories of causal reasoning and/or causal Bayes nets were supported by the empirical evidence on lay theories of depression. There is, however, a considerable lack of evidence with respect to two crucial aspects. First, the interplay between higher-order theories of causation and illness and causal models of specific conditions like depression has not been investigated. Second, the influence of causal beliefs on categorization and reasoning has not been explored and the evidence with respect to decision making is still scarce, especially in non-Western cultural groups.

## Discussion

The present paper explored how cognitive psychological theories of causal learning and reasoning can inform research on systems of (causal) beliefs in different cultural groups. Based on causal model theories (Waldmann, 1996; Sloman, 2005) and causal Bayes net theories (Pearl, 2000; Glymour, 2001; Griffiths and Tenenbaum, 2009; Tenenbaum et al., 2011) predictions for systems of causal beliefs were derived. These predictions were applied to lay theories of depression. Lay theories of depression seemed to be an appropriate test case, as depression is present globally with a substantial prevalence and a common core of somatic and psychological symptoms. Established causes include both observable and non-observable factors. Therefore, all derived predictions could be tested. Most predictions entailed a similarity between different cultural groups. Differences in beliefs were only expected with respect to higher-order theories and inferred hidden causes. It was also predicted that causal beliefs should affect the categorization and diagnosis of depression as well as preferences and decisions with respect to treatment.

A systematic literature review on lay theories of depression was conducted and eligible papers were analyzed systematically by classifying assumed causes and preferred treatments into common categories. Results showed that members of all investigated cultural groups held causal beliefs about generic causes of depression and that beliefs constituted complex causal models. As predicted, substantial agreement was found between different cultural groups with respect to easily observable causes of depression, that is, stress due to environmental factors like marital problems and psychological variables like depressive thinking styles. Less agreement resulted for hidden causes. Substantial differences were found with respect to supernatural causes between Western and non-Western cultural groups and between different non-Western groups. Many of these beliefs seemed to be culture-specific (e.g., the role of karma or the influence of ancestral spirits). Assumptions about these causes also seemed to be informed by higher-order theories of causation and illness, although none of the reviewed studies directly investigated this relation empirically.

The usage of causal beliefs in reasoning and decision making has rarely been explored systematically. Especially evidence from non-Western cultural groups is lacking. When investigated, rather good agreement between causal beliefs and treatment preferences were found for Western cultural groups. The few results for non-Western groups appear to be mixed. It seems that other factors apart from causal beliefs may have an important impact on treatment preferences as well.

Taken together, the results tend to support the derived predictions. This indicates that cognitive psychological theories of causal learning and reasoning can be used to derive testable predictions for systems of causal beliefs.

### Limitations

There are a number of limitations that need to be pointed out. First, there are limitations concerning the systematic literature review. Only publications describing original research on causal beliefs about depression were included. In addition, studies had to present quantitative results. We deliberately constrained ourselves to quantitative studies in order to be able to rank order causes for importance. Purely qualitative studies were therefore excluded. In consequence, more studies on non-Western cultural groups were excluded than studies on Western cultural groups. This is particular unfortunate as—for example—studies investigating cultural groups in Iran (Dejman et al., 2010), Uganda (Okello and Ekblad, 2006), and Vietnam (Niemi et al., 2009) were not considered. The same is true for studies looking at different religious groups living in the same country (e.g., Loewenthal and Cinnirella, 1999). However, these studies reported similar findings as the studies reviewed here. One exception seems to be that religious or spiritual people tended to believe more strongly in supernatural causes (e.g., loss of faith) and endorsed respective practices for treatment in both Western and non-Western groups (Wittink et al., 2009).

Studies included in this review were published in medical, medical-anthropological, social science and psychology journals. We cross-checked reference lists for further relevant publications. We are not aware of missing important empirical, quantitative studies published elsewhere. Despite this effort, hardly any anthropological studies ended up in this review. This is probably due to our focus on quantitative studies. Another reason is that the searched databases only encompass a few journals publishing anthropological research, although *Medical Anthropology*, *Transcultural Psychiatry*, and *Social Science and Medicine* seem to be the major outlets for work on lay concepts and theories of depression and other mental illnesses. A third reason may be that we concentrated on a specific mental disorder. It might well be that anthropologists take a broader perspective and look at theories of mental illness or mental distress instead of particular diseases (e.g., Kleinman, 1980; Kirmayer and Valaskakis, 2008). The reason, however, may be more fundamental. Beller et al. (2012) pointed out that cognitive science and anthropology might be incompatible with respect to perspective and methods. Therefore, findings from anthropology may be difficult to use to test predictions derived from cognitive psychological theories.

Second, there are limitations concerning the methodological rigor of the reported studies. All studies reviewed here systematically assessed participants' causal beliefs, which is good. But not all studies presented participants with case descriptions of depression. Therefore, it is not clear that all participants had the same understanding of the term “depression.” Some of the non-Western cultural groups lived in Western countries, which may have changed their beliefs about depression. In fact, some Yoruba people pointed out that they would give different answers depending on whether they were in the UK or Nigeria (Lavender et al., 2006). Hence differences between Western and non-Western cultural groups may be underestimated. Unfortunately, the relation between assumed causes and treatment preferences were only assessed in limited number of studies. Found correlations were generally low to moderate. Error accumulation due to the large number of statistical tests was almost never taken into account. Hence, the statistical validity of the results has to be rated as rather moderate.

Finally, we only tested the predictions derived from cognitive-psychological theories with respect to causal beliefs on depression. It might be that lay theories of depression are different from other lay theories. Although we cannot exclude this possibility, our results seem to be in line with lay theories about other topics (cf. Furnham, 1988). Nevertheless, more evidence on other systems of causal beliefs is needed to corroborate the present findings.

### Implications for future research

The review identified two areas of research, which merit further attention by researchers investigating causal cognition in different cultural groups. One area is the interplay between higher-order theories and causal models for particular issues. Higher-order theories include general notions of causation and general theories of a domain (e.g., lay theories of illness). Although more general lay theories have been investigated (e.g., Furnham, 1988), there is little research on how theories on different levels of abstraction interact with each other and the observable evidence in different cultural groups. Hierarchical Bayes nets (Tenenbaum et al., 2011) allow us to derive specific predictions for the causal models people will induce from a set of observations and respective higher-order theories. In order to conduct respective experimental studies a multi-method approach seems to be advisable. A triangulation approach (Atran and Medin, 2009) would allow us to properly investigate the influence of different cultural backgrounds on higher level theories. For example, theories of skin diseases could be assessed in Western and Indian groups in the UK and in India. This way country and cultural background could be disentangled. In a research study, first higher level theories could be assessed using interviews. Respective methodologies have already been developed in anthropology and cultural psychology (e.g., Kleinman, 1980; Weiss et al., 1992; Atran and Medin, 2009). Based on the interviews, lay theories could be reconstructed on the group and the individual level. In a second step, participants in the study could be confronted with a series of cases showing a new, previously unknown medical condition. Dermatological problems seem to be a good starting point as there are many forms. Hence new forms can be created easily without violating general expectations. In addition, dermatological problems have many different causes (e.g., allergic reactions, cancer, somatization problems). Like in the case of depression, some of these causes are directly observable, while others are hidden. This would allow researchers to manipulate the data presented to participants. Data can be presented as descriptions of individual cases, which would ease understanding. Data may show a contingency between an observable cause (e.g., a new type of clothing) and the condition to be explained (e.g., itchy dark purple spots in the arm pit, which start to bleed later on) or the observable causes may be unrelated to the symptoms. After hearing (or reading) about a series of cases, participants would be asked to explain either a typical single case or to provide a generic explanation of the problem. Hence participants would be asked about their theory of the illness on a token (specific single case) and a type level (generic model). One prediction to be tested would be that explanations are more strongly influenced by the observed data when observable causes were related to the condition, but more strongly affected by higher level theories of illness and skin problems when there were no contingent observable causes. In addition to experimental research, real world test cases could be explored. An interesting, historic test case may be people's causal beliefs about AIDS when the respective syndrome first grabbed the public's attention. Another example is bovine spongiform encephalitis (BSE, mad cow's disease) and variant Creuzfeldt-Jakob Disease (vCJD, the human version of BSE), which also initially created a puzzle for experts and lay-people. In both cases it is predicted that people resorted to higher-order theories about illness to account for the observed syndrome.

The second area of interesting future research concerns the usage of causal beliefs for categorization, diagnosis, prognosis, and decision making. There is already some evidence that folk ecological causal beliefs affect categorization and decision making (cf. Atran and Medin, 2009), but more evidence from different domains and different cultures would be interesting. Experimental and non-experimental research could provide important insights. While non-experimental research would show whether decisions and judgments are coherent with causal beliefs, experimental research could show whether, when and how causal beliefs affect judgments and decisions. Cognitive psychology provides a wealth of experimental paradigms to study causal reasoning in experts and lay-people in a rigorous manner. Such experimental research allows us to distinguish between judgments and decisions that are merely recalled from memory and judgments and decisions that are based on reasoning. When a decision can be recalled from memory, because it had been taken under the same circumstances before, no causal reasoning is necessary. Only when no judgment or decision is known right away, causal reasoning based upon pre-existing causal beliefs and the observed situation may become relevant (cf. Sloman and Hagmayer, 2006; Hagmayer and Sloman, 2009). Hence, if we want to study causal reasoning of lay-people in everyday contexts, we need to create novel, but meaningful scenarios, in which they can resort to their causal knowledge, but do not necessarily have to. The work by Kim and Ahn (2002) is a good example for well controlled experimental research. In these studies, participants' causal beliefs about mental illnesses were assessed individually before they were confronted with novel judgment and decision problems, which were created based on their idiosyncratic causal beliefs. For example, participants were asked to diagnose new patients, which conformed to different degrees to the causal assumptions held by the individual participant. One may argue, however, that the materials presented to participants in these studies were still impoverished in comparison to real life complexity. This is true, but the basic paradigm could be extended respectively. Using well established interview techniques (e.g., Kleinman, 1980; Atran and Medin, 2009), explanatory models of—for example—particular mental or somatic diseases could be assessed. Based on these models, different and complex case vignettes could be created. These case vignettes could either describe prototypical cases, which show all expected symptoms, or cases, which show only a subset of symptoms. In addition, it could be manipulated how many potential causes and/or risk factors of the condition are present. As well, the course of the condition (its development over time) could be more or less typical. After creating the cases, it would be important to assess how familiar participants are with these cases and how often they had heard about a respective diagnosis and treatment before. This would indicate whether participants could resort to their memory or would have to engage in reasoning. Based on the case vignettes three types of dependent variables could be collected. First participants could be asked to name the patient's problem. This would show how participants would categorize the case. To collect quantitative data, participants could be requested to rate how likely the person has the respective condition. Second, participants could be asked to explain the condition of the respective patient. Hence, they would be asked to engage in diagnostic causal reasoning. Again, ratings of potential causes could be collected as a quantitative measure. Third, participants could be asked to choose a course of action, that is, they would have to decide on a treatment. As before, quantitative ratings of different treatments could be requested.

The research strategies outlined in the previous two paragraphs combine elements from cognitive psychological, cultural psychological and cognitive anthropological research. This shows that these approaches are not incommensurate (Unsworth, 2012). Anthropological research does not only provide interesting research questions (Whitehouse and Cohen, 2012), but also methods to develop a deep understanding of the beliefs held by people in different cultural groups as well as the inferences and decisions that these people are likely to make (Astuti and Bloch, 2012). Existing ethnographic research may already provide descriptions of higher level theories, which are needed to conduct the type of research proposed here. Murdock's work (1980) is an excellent example in this regard.

## Conclusion

Research on systems of beliefs in different cultural groups and cognitive psychological research on causal learning and reasoning *can* inform each other. In our view, they *should* inform each other. In this paper, we derived predictions from cognitive-psychological theories for systems of lay causal beliefs. Social science and cognitive anthropological research can and—to some degree—already does provide empirical results to test these predictions in different cultural groups. Moreover, in order to investigate causal learning and reasoning in everyday contexts, it is necessary to know which causal beliefs people may bring to bear when they are confronted with judgment and decision making tasks. These beliefs range from abstract notions of causality to causal models for particular issues. In addition, it is important to know about other beliefs like moral convictions, which may also affect causal judgments and decisions on actions (Liu and Ditto, 2013). Only when these beliefs are known, it will be possible to study the interplay of causal beliefs and causal reasoning in everyday life through experimental research.

### Conflict of interest statement

The authors declare that the research was conducted in the absence of any commercial or financial relationships that could be construed as a potential conflict of interest.
